# Supplementary material for: Transcriptomic Profiling for the Autophagy Pathway in Colorectal Cancer
Source: Int J Mol Sci. 2020 Sep 26;21(19):7101. doi: 10.3390/ijms21197101 (PMC7582824; doi:10.3390/ijms21197101)
Supplement: Supplementary file 1 [file ijms-21-07101-s001.zip › Supplementary table 4_220920.doc]

| **Algorithm** | **Dataset** | **Target genes** | **Aim** |
| --- | --- | --- | --- |
| **EGSEA** | RNAseq CRC | All protein coding | Evaluate general importance of autophagy in CRC |
| **COMMUNAL** | RNAseq CRC, array CRC and celline CRC | 46 selected autophagy-related genes | Assess whether there are CRC clusters differing in terms of expression of selected autophagy genes |
| **t-test or Kruskal-Wallis test** | RNAseq CRC, array CRC |  | To compare import ant molecular and clinical variables between autophagy clusters |
| **sPLS-DA** | RNAseq CRC, array CRC and celline CRC | 46 selected autophagy-related genes | To find autophagy-related genes that best separate “autophagy clusters” |
| **DE** (differential expression) | RNAseq CRC, array CRC | All protein coding | To present differences/similarities in gene expression of 46 autophagy genes between autophagy clusters |

Supplementary Table 4. The table summarizes statistical  procedures utilized in the current study.
